# Neuropeptide Y Knockout Mice Reveal a Central Role of NPY in the Coordination of Bone Mass to Body Weight

**DOI:** 10.1371/journal.pone.0008415

**Published:** 2009-12-22

**Authors:** Paul A. Baldock, Nicola J. Lee, Frank Driessler, Shu Lin, Susan Allison, Bernhard Stehrer, En-Ju D. Lin, Lei Zhang, Ronald F. Enriquez, Iris P. L. Wong, Michelle M. McDonald, Matthew During, Dominique D. Pierroz, Katy Slack, Yan C. Shi, Ernie Yulyaningsih, Aygul Aljanova, David G. Little, Serge L. Ferrari, Amanda Sainsbury, John A. Eisman, Herbert Herzog

**Affiliations:** 1 Osteoporosis and Bone Biology Program, Garvan Institute of Medical Research, St Vincent's Hospital, Sydney, Australia; 2 Neuroscience Program, Garvan Institute of Medical Research, St Vincent's Hospital, Sydney, Australia; 3 Department of Physiology and Biophysics, Georgetown University Medical Center, Washington, D. C., United States of America; 4 Department of Orthopaedic Research and Biotechnology, The Children's Hospital at Westmead, Sydney, Australia; 5 Department of Molecular Medicine and Pathology, University of Auckland, Auckland, New Zealand; 6 Service and Laboratory of Bone Diseases, Department of Rehabilitation and Geriatrics, Faculty of Medicine, Geneva University Hospital, Geneva, Switzerland; 7 School of Medical Sciences, University of New South Wales, Sydney, New South Wales, Australia; 8 Faculty of Medicine, University of New South Wales, Sydney, New South Wales, Australia; The University of Queensland, Australia

## Abstract

Changes in whole body energy levels are closely linked to alterations in body weight and bone mass. Here, we show that hypothalamic signals contribute to the regulation of bone mass in a manner consistent with the central perception of energy status. Mice lacking neuropeptide Y (NPY), a well-known orexigenic factor whose hypothalamic expression is increased in fasting, have significantly increased bone mass in association with enhanced osteoblast activity and elevated expression of bone osteogenic transcription factors, Runx2 and Osterix. In contrast, wild type and NPY knockout (*NPY ^−/−^*) mice in which NPY is specifically over expressed in the hypothalamus (AAV-NPY+) show a significant reduction in bone mass despite developing an obese phenotype. The AAV-NPY+ induced loss of bone mass is consistent with models known to mimic the central effects of fasting, which also show increased hypothalamic NPY levels. Thus these data indicate that, in addition to well characterized responses to body mass, skeletal tissue also responds to the perception of nutritional status by the hypothalamus independently of body weight. In addition, the reduction in bone mass by AAV NPY+ administration does not completely correct the high bone mass phenotype of *NPY ^−/−^* mice, indicating the possibility that peripheral NPY may also be an important regulator of bone mass. Indeed, we demonstrate the expression of NPY specifically in osteoblasts. In conclusion, these data identifies NPY as a critical integrator of bone homeostatic signals; increasing bone mass during times of obesity when hypothalamic NPY expression levels are low and reducing bone formation to conserve energy under ‘starving’ conditions, when hypothalamic NPY expression levels are high.

## Introduction

Bone remodelling has traditionally been viewed as an endocrine- and paracrine-regulated process. This view is rapidly changing, however, with growing evidence demonstrating that neuronal factors are critical for normal bone homeostasis. Moreover, efferent neural pathways from the hypothalamus potently modify the activity of bone cells. Studies in leptin-deficient (*ob/ob*) mice revealed that the adipokine leptin, in addition to its actions as an important circulating indicator of the level of adiposity, also modulates bone formation through activation of central, hypothalamic relays via efferent sympathetic nervous output which directly modulates osteoblast activity [1]–[3]. A major target of leptin in the hypothalamus is the neuropeptide Y (NPY) system, one of the most prominent regulators of appetite and energy homeostasis [4]. Leptin down-regulates NPY expression in the hypothalamus and in this way reduces appetite and normalises energy expenditure [5]. Importantly however, the NPY system has also been shown to regulate bone homeostasis [6]. Amongst the 5 known Y-receptors (Y1, Y2, Y4, Y5 and in certain species also Y6) [7], [8], NPY mediates its effects on energy homeostasis via hypothalamic Y1 and Y2 receptors [6]. Interestingly, these Y-receptors have also been reported to be critical in the regulation of bone homeostasis with the specific deletion of the Y2-receptor in the hypothalamus resulting in a bone anabolic phenotype [6].

However, the actions of the NPY system in bone are more complex than a simple downstream mediator for leptin. Specifically, Y2-mediated changes occur consistently throughout the skeleton in these mice, while alterations in leptin levels induce opposing effects on cortical and cancellous bone, as evident in studies involving Y2^−/−^;*ob/ob* double mutant mice [9]. Furthermore, Y1 receptors have been identified on osteoblasts and a lack of Y1 receptors in mice also leads to increased bone mass [10]. The Y1-mediated effect on bone is not dependent on hypothalamic Y1 receptors suggesting an additional direct action of the NPY system on bone homeostasis [6], [8], [11].

Despite the demonstrated actions of NPY, Y receptors in the control of bone homeostasis, the role of NPY in this process is yet to be defined. Although NPY is the predominant ligand in the central nervous system, Y receptors can also be activated by the two other family members, peptide YY (PYY) and pancreatic polypeptide (PP). Possible redundancy in the functions of NPY-like ligands may explain why initial examination of NPY deficient mice suggested no skeletal changes [12], a finding contradictory to experiments showing significant increases in bone mass following loss of NPY-producing neurons from the arcuate nucleus of the hypothalamus using monosodium glutamate treatment by the same group [13]. Therefore we used a systematic approach to investigate the specific role of NPY signalling on bone homeostasis employing several NPY mutant mouse models including specific re-introduction of NPY into the hypothalamus of otherwise NPY deficient adult mice.

## Results

### High Bone Mass and Reduced Fat Mass in NPY ^−/−^ Mice

**Figure 1:**
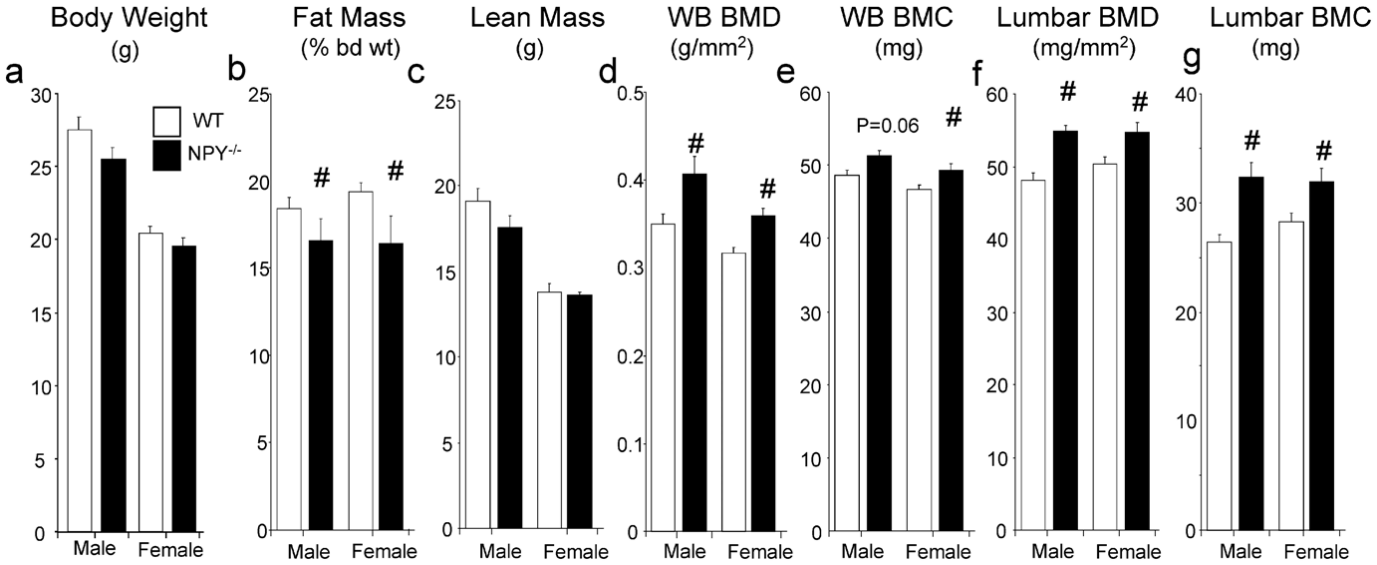
Generalized bone anabolic phenotype in 4 month old male and female *NPY ^−/−^* mice. (a) Body weight. Dual X-ray absorptiometry analysis of (b) fat mass, (c) lean mass indicate changed energy homeostasis in *NPY ^−/−^* mice. Bone mass and density was also greater as seen in (d) whole body BMD, (e) whole body BMC, (f) lumbar BMD and (g) lumbar BMC. n = 8−14, data expressed as mean±SE.

While there was no effect of NPY ablation on body weight (Figure 1a), body fat mass was significantly reduced in *NPY ^−/−^* compared to wild type mice, as evidenced by decreased whole body fat mass measured by DXA (Figure 1b), with no effect of genotype on lean body mass (Figure 1c). Importantly, lack of NPY was associated with a greater whole body BMD as well as a greater whole body BMC apparent in both genders, although the increase in BMC only reached significance in female *NPY ^−/−^* (Figure 1d, 1e). Consistent with the whole body measurement, lumbar BMD and BMC were significantly increased in *NPY ^−/−^* mice compared to gender-matched wild type controls (Figure 1f, 1g).

### Greater Cortical Bone Formation in NPY ^−/−^ Mice

**Figure 2:**
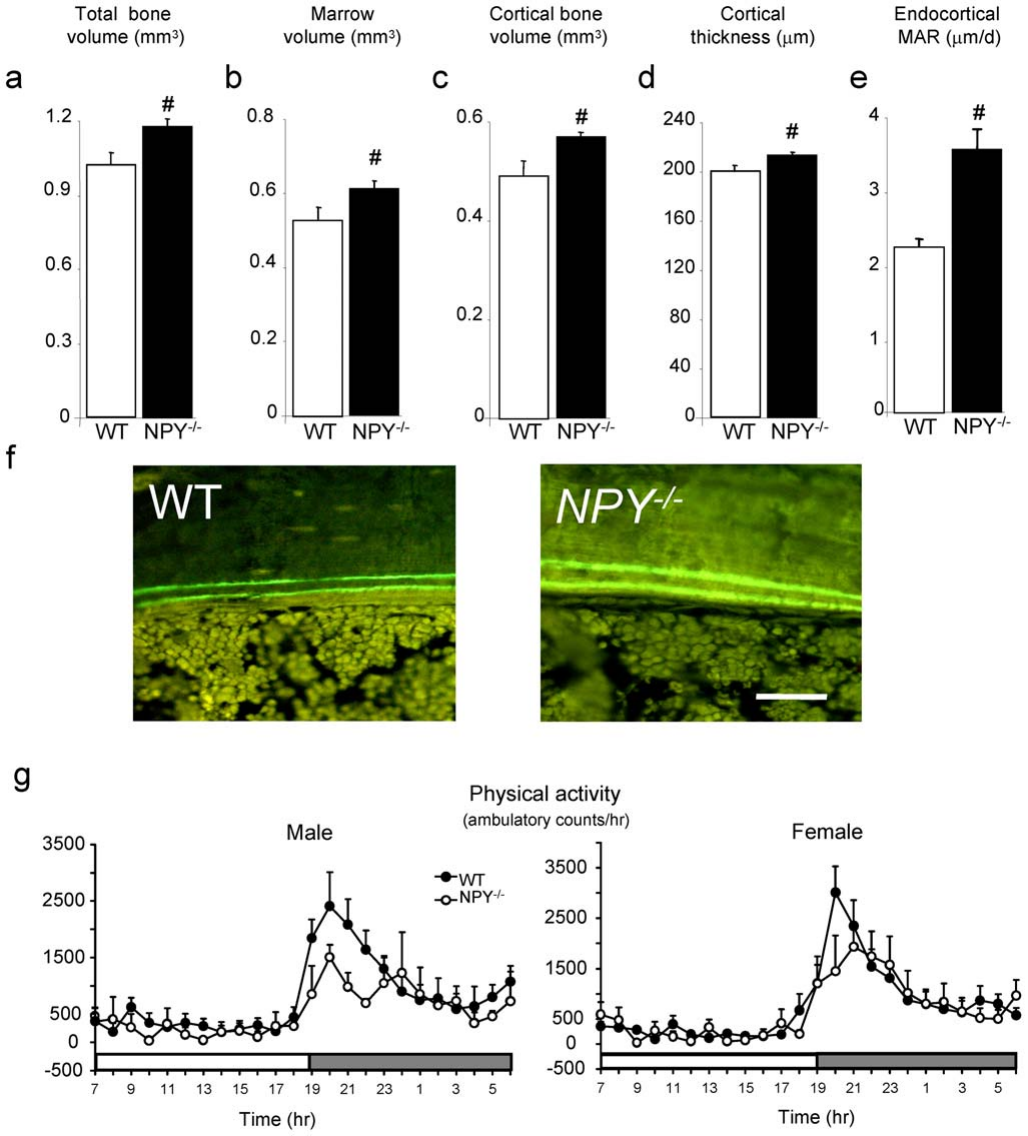
Greater cortical bone in *NPY ^−/−^* mice. Peripheral quantitative computed tomography of mid-femur in male mice showing (a) total bone volume, (b) marrow volume, (c) cortical bone volume and (d) cortical thickness. (e) Mid femoral endocortical mineral apposition rate, (f) photomicrograph of endocortical bone formation. (g) 24 hour home cage activity. Scale bar represents 50 µm. # p<0.05 vs wild type. n = 8−14, data expressed as mean±SE.

In order to evaluate the DXA results, cortical bone was examined by peripheral quantitative tomography. There was a significant increase in cortical bone size in *NPY ^−/−^* mice. Total bone volume and marrow volume of the mid-femur of *NPY ^−/−^* mice were both increased, resulting in greater cortical bone volume and cortical thickness in *NPY ^−/−^* mice compared to wild type (Figure 2a–2d). These changes were associated with enhanced cortical bone formation, as shown by elevated endocortical mineral apposition rate (MAR) (Figure 2e, 2f). Importantly, these changes in cortical bone mass and formation were evident in the absence of any increases in motor activity in either male or female *NPY ^−/−^* mice, negating a potential role of activity and weight bearing in the increases in bone mass seen in these mice (Figure 2g). Indeed, a trend for reduced physical activity was evident in *NPY ^−/−^* mice in the dark phase, but this was not significant, nor was it seen in the overall 24-hour period (Figure 2g).

### Generalized Increase in Bone Formation in NPY ^−/−^ Mice

**Figure 3:**
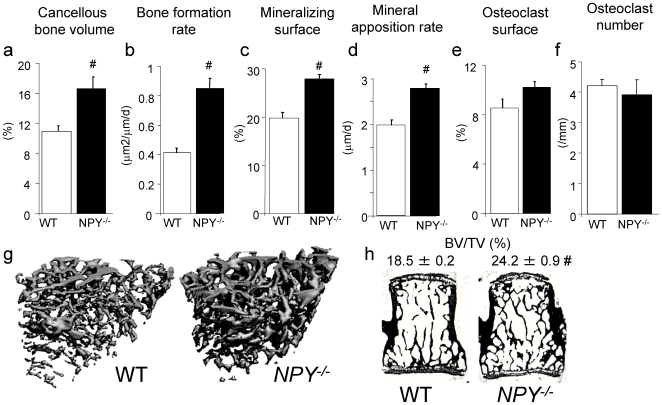
Greater cancellous bone volume and formation in *NPY ^−/−^* mice. Histomorphometric analysis of distal femoral metaphysis in male mice showing (a) cancellous bone volume, (b) bone formation rate, (c) mineralizing surface, (d) mineral apposition rate, (e) osteoclast surface and (f) osteoclast number. (g) Representative micro-computed tomographs of the distal femoral metaphysis of 4 month old male wild type and *NPY ^−/−^* mice. (h) 4th lumbar vertebral sections from male wild type and *NPY ^−/−^* mice, displaying greater cancellous bone volume (BV/TV) in mutant mice. # p<0.05 vs wild type. n = 8−14, data expressed as mean±SE.

In the distal femoral metaphysis, cancellous bone volume was greater in *NPY ^−/−^* mice, coincident with a 2-fold increase in bone formation rate compared to that in wild type mice (Figure 3a, 3b). This change was associated with an increase in the extent (mineralizing surface) and speed (mineral apposition rate) of bone formation (Figure 3c, 3d). Bone resorption indices in *NPY ^−/−^* mice – i.e. osteoclast surface and number – were not significantly changed (Figure 3e, 3f), demonstrating that NPY preferentially controls the anabolic aspects of bone homeostasis. Femoral data is shown for male mice, but results were similar in females. The effects of NPY deletion on femoral cancellous bone were also evident in micro computed tomographs of the distal femoral metaphysis (Figure 1g) and cancellous bone volume of the 4^th^ lumbar vertebrae (Figure 1h), indicating a generalized anabolic effect of NPY deletion throughout the skeleton.

### Elevated Hypothalamic NPY Expression Reduces Bone Formation

**Figure 4:**
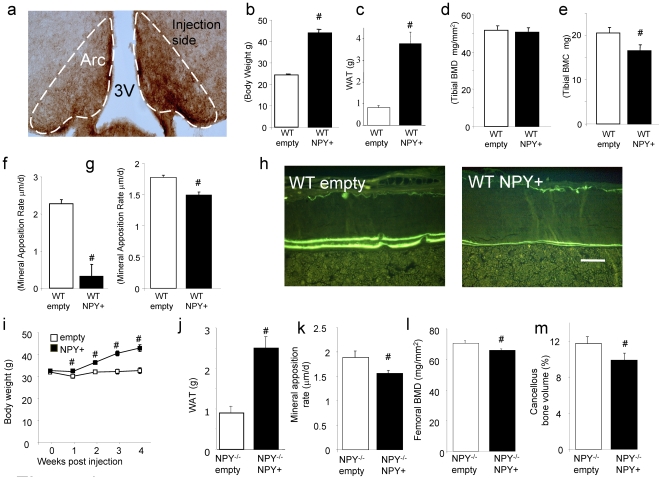
Hypothalamic NPY over-expression inhibits bone formation, despite increased body weight. Photomicrograph of coronal brain section showing NPY over expression following unilateral rAAV-NPY+ injection in the arcuate nucleus (Arc). Changes in (b) body weight, (c) white adipose tissue, (d) tibial BMD, (e) tibial BMC, (f) mid femoral endocortical and (g) mid femoral periosteal mineral apposition rate in response to hypothalamic NPY over expression in wild type mice (WT NPY+) compared to control (WT empty). (h) Representative photomicrographs of femoral endocortical mineral apposition rate WT empty or WT NPY+ mice. Changes in (i) body weight, (j) fat mass, (k) distal femoral metaphyseal mineral apposition rate, (l) femoral BMD, and (m) distal femoral metaphyseal cancellous bone volume in germline *NPY ^−/−^* mice after hypothalamic NPY over expression (NPY ^−/−^ NPY+) compared to controls (NPY ^−/−^ empty). # p<0.05 vs empty. Scale bar represents 20 µm. n = 7−12, data expressed as mean±SE.

To investigate whether alterations in central NPY levels are responsible for the anabolic bone phenotype seen in *NPY ^−/−^* mice, NPY was over-expressed specifically in the hypothalamus of adult wild type mice. Wild type mice were injected unilaterally with a recombinant adenovirus-associated virus producing a localized increase in NPY expression (rAAV-NPY+) in the hypothalamus, targeting the arcuate nucleus (Arc) (Figure 4a), the brain region critical in mediating the central effects of Y receptor signaling on both energy balance and bone mass [6], [8]. Four weeks post-injection, body weight was significantly increased in wild type rAAV-NPY+-injected animals (WT NPY+) compared to wild type controls, injected with an empty virus (WT empty) (Figure 4b). This weight gain was associated with an increase in white adipose tissue mass (Figure 4c).

Given the strong and positive association between increases in body weight and bone mass, in part due to mechanical effects of weight bearing [14], it is notable that tibial BMC was reduced by 19% (p<0.05) in WT NPY+ mice compared to WT empty controls over the 4-week period, with no change in tibial BMD (Figure 4d, 4e). This NPY-mediated bone loss was due to the specific action of NPY in the hypothalamus, as no changes in bone homeostasis were evident following rAAV-NPY+ injection into the hippocampus [8]. Histological examination of bones revealed that elevation of hypothalamic NPY expression decreased cortical osteoblast activity, with a 7-fold reduction in endosteal MAR and a significant reduction in periosteal MAR in WT NPY+ mice compared to WT empty controls (Figure 4f, g). These changes can be clearly seen on the endosteal surface after injection of dual fluorescent labels (Figure 4h), demonstrating the powerful action of hypothalamic-NPY activated pathways on inhibiting osteoblast activity.

In order to determine whether the bone anabolic phenotype observed in *NPY ^−/−^* mice is wholly due to the lack of hypothalamic NPY signaling, an additional transgenic mouse model was generated where NPY expression was restricted solely to the hypothalamus. This model was achieved by re-introducing NPY specifically into the hypothalamus of *NPY ^−/−^* mice via stereotactic injection of rAAV-NPY+ (*NPY ^−/−^* NPY+). As shown in Figures 4i and 4j, *NPY ^−/−^* NPY+ -mice had a greater body weight gain and more than doubled adiposity within 4 weeks compared to *NPY ^−/−^* empty mice, confirming the viability of the NPY reintroduction procedure and demonstrating the critical role hypothalamic NPY plays in regulating body weight and fat mass. Interestingly however, the elevated bone mass and bone formation seen in *NPY ^−/−^* mice was only partially corrected by hypothalamic re-introduction of NPY in otherwise NPY deficient mice. As shown above, global deletion of NPY led to a 40% increase in MAR in femoral cancellous bone compared to wild type controls (Figure 3d). However, restoration of NPY expression in the hypothalamus of *NPY ^−/−^* mice resulted only in a partial normalization of MAR to a level approximately 20% lower then *NPY ^−/−^* mice, with similar reductions in the change in femoral bone mass and cancellous bone volume (Figure 4k–4m). This inability of hypothalamic NPY to completely restore a normal bone phenotype in *NPY ^−/−^* mice suggests that non-hypothalamic pathway(s) may also be important in NPY mediated bone homeostasis.

### NPY Signalling on Bone Cells Alters Osteogenesis In Vivo and In Vitro

**Figure 5:**
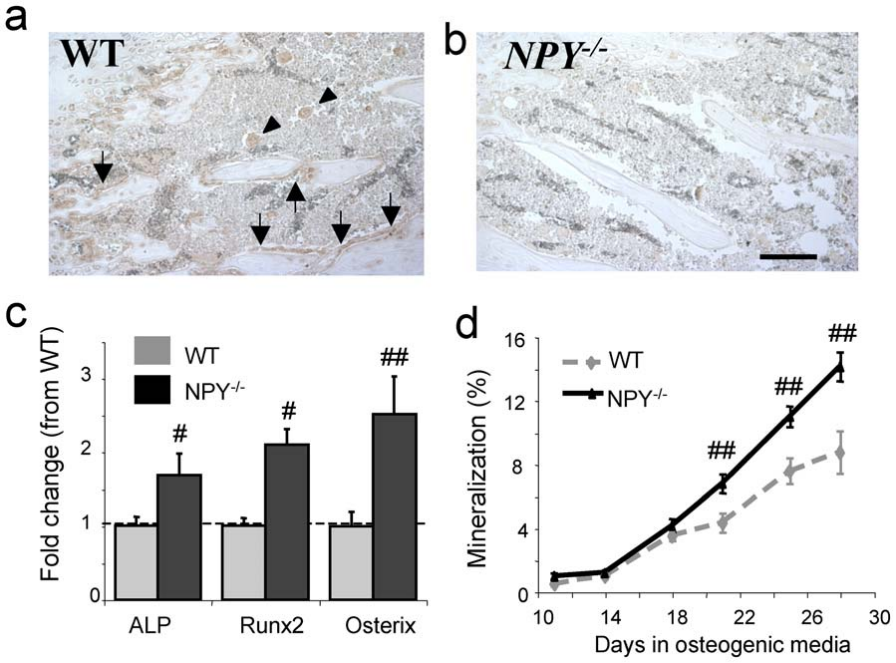
NPY expression and action in bone tissue. (a) Analysis of NPY expression by *in situ* hybridisation in wild type mice shows specific staining of osteoblasts employing a NPY antisense probe in cancellous and cortical bone (arrows) and megakaryocytes (arrowheads). (b) This staining is absent in *NPY ^−/−^* mice. (c) Levels of ALP, Runx2 and Osterix are greater in *NPY ^−/−^* mice compared to wild type as determined by quantitative real-time PCR on mRNA from long bones. (mean±SE. n = 5−6 mice per genotype.). (d) Bone marrow stromal cells isolated from *NPY ^−/−^* mice produce more mineral *in vitro* than wild type mice. (mean±SE, n = 10−15 wells per time, 3 independent experiments). # p<0.05, ## p<0.01 vs wild type, scale bar represents 50 µm.

In order to clarify whether NPY also regulates bone homeostasis by direct action on bone cells and whether NPY is expressed in bone tissue itself, bone sections were examined by *in situ* hybridisation. Both cancellous and cortical osteoblasts from wild type mice stained positively for NPY mRNA (Figure 5a) with no staining in *NPY ^−/−^* bone tissue (Figure 5b), providing the first evidence of NPY expression in osteoblasts *in vivo*.

To further investigate what role NPY may play in osteoblast function and development, mRNA was isolated from the bones of wild type and *NPY ^−/−^* mice and analysed for the expression of key osteogenic markers. In keeping with their high bone mass and anabolic phenotype, mRNA expression of alkaline phosphatase (ALP) was significantly up-regulated in the long bones of *NPY ^−/−^* mice (Figure 5c). Importantly, there was also an up-regulation of both Runx2, and Osterix mRNA levels in the *NPY ^−/−^* mice (Figure 5c). Moreover, *in vitro* osteoblast activity was enhanced in the absence of NPY, evident by a greater time-dependent increase in mineralisation by *NPY ^−/−^* bone marrow stromal cells under osteogenic conditions than cells isolated from wild type mice (Figure 5d).

Taken together, these data indicate that global lack of NPY induces effects inherent to osteoblasts in both cortical and cancellous bone and in the apical and appendicular skeletons. Given that NPY is expressed in osteoblasts, these data further support the concept that NPY could be a direct local regulator of osteoblast function, in addition to effects mediated via the hypothalamus, and highlights the fact that NPY may act via neuronal as well as autocrine mechanisms to regulate bone homeostasis.

## Discussion

This study has identified a powerful inverse relationship between NPY signaling in the hypothalamus and bone formation. A generalized bone anabolic response resulting from loss of NPY signaling was evident throughout the skeleton, including cortical and cancellous bone as well as axial and appendicular sites. In contrast, NPY over expression in the hypothalamus resulted in an inhibition of bone formation. This central action is consistent with signaling via Y2 receptors expressed on neurons of the arcuate nucleus, as is evidenced by the opposing response reported following specific deletion of Y2 receptors from this region of the hypothalamus [6]. Thus NPY acts in the hypothalamus to tonically inhibit bone formation, and likely does so through signals transduced by Y2 receptors.

**Figure 6:**
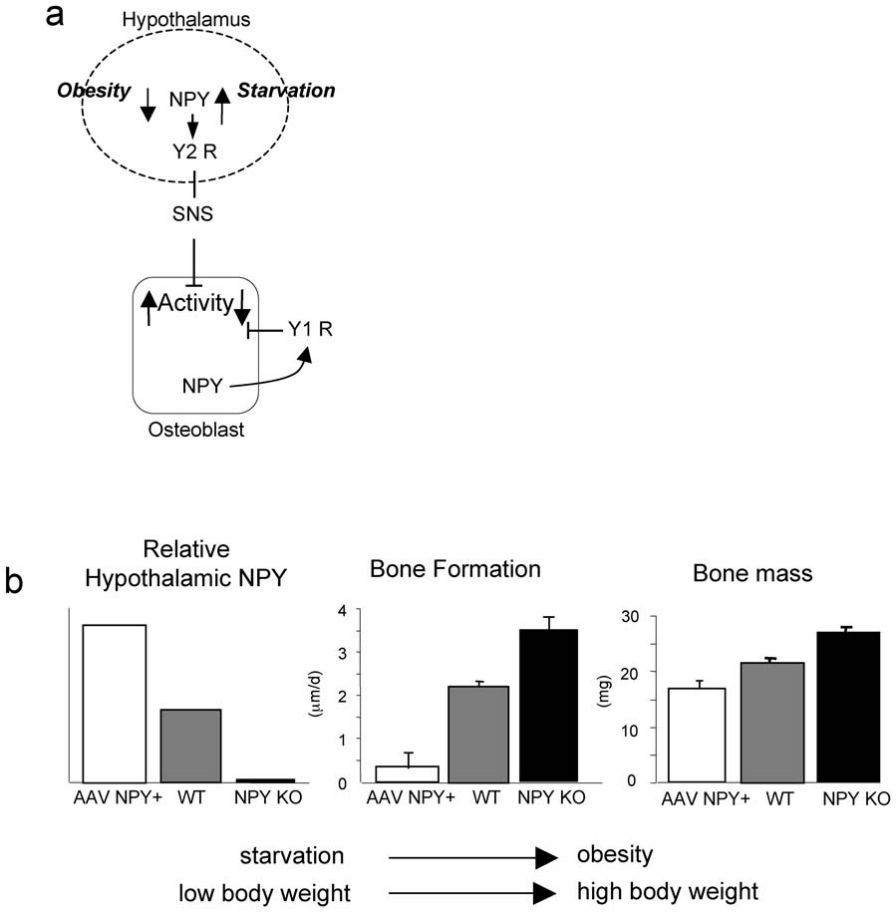
Schematic representation of the co-ordinate regulation of body weight and bone by NPY. (a) Altered energy homeostatic demand regulates hypothalamic NPY production, which signals via arcuate Y2 receptors through efferent sympathetic relays to the osteoblast. At the osteoblast, tonic cell activity is modulated by exogenous neural inputs as well as local NPY production, acting through cell surface Y1 receptors. (b) NPY exhibits an inverse relationship with both energy homeostasis and bone mass. As body weight increases as a result of positive energy homeostasis, the decrease in NPY signaling stimulates the production of bone to match body weight to bone mass. Likewise, negative energy homeostasis, and the coincident reduction of body weight is accompanied by increased NPY, which inhibits the production of bone, thereby conserving energy and increasing the mobilization of nutrient stores form bone.

Results from this study suggest that in addition to the well defined actions of loading from body weight on bone mass, the central ‘perception’ of energy status may have an important influence on bone mass. This is particularly evident in rAAV-NPY+ injected mice. The viral promoter controlling NPY production prevents the normal feedback by signals such as increased serum leptin from inhibiting hypothalamic NPY expression [12], [15]. As a result, the “starvation” signal is not attenuated and body weight continues to increase. Importantly, despite the marked increase in body weight caused by this experimental condition, a decrease in bone mass occurred. While the decrease in bone formation in these mice may in part also result from reduced physical activity [16] and thereby altered weight bearing [17], this result is also consistent with a skeletal response to the starvation signal induced by AAV NPY+ [18]. Thus the central perception of body weight may be important to the regulation of bone mass, in addition to the actual mechanical forces in the bone tissue itself. In this manner, during weight loss, increasing NPY ensures calories are not wasted on the production/maintenance of unneeded skeletal tissue, thereby also increasing the supply of minerals and nutrients stored in skeletal tissue for other vital processes. Conversely, during periods of high nutrient intake and resultant weight gain, reduction in NPY expression ensures sufficient bone formation to guarantee mechanical competence of the skeleton during the period of increased weight bearing. Importantly, we showed here that reduced NPY levels in NPY knockout mice are not associated with significant changes in activity levels, indicating the skeletal response to NPY are not activity-dependent. NPY is therefore ideally placed to match bone mass to body weight, a major regulatory influence on bone homeostasis [19] (Figure 6b).

Central circuits alone, however, do not appear to explain the entire NPY-mediated signaling pathway to bone, as restoration of hypothalamic NPY expression in *NPY ^−/−^* mice failed to completely normalize osteoblast activity. This incomplete rescue of the *NPY ^−/−^* phenotype by central NPY replacement indicates a potential role for non-hypothalamic NPY pathways. This finding is consistent with a Y1 receptor-mediated action in the periphery, since hypothalamus-specific deletion of Y1 receptors did not replicate the greater bone formation evident in germline *Y1^−/−^* mice [10]. This opens the possibility that lack of NPY in osteoblasts may have contributed to the phenoptype of *NPY ^−/−^* mice, consistent with our demonstration of NPY expression in osteoblasts, altered expression of osteogenic transcription factors in bone as well as increased *ex vivo* mineralization of osteoblasts from *NPY ^−/−^* mice. Indeed, identification of NPY expression in the osteoblast, along with local expression of Y1 receptor [10], [20] indicates the potential for local paracrine/autocrine control of osteoblast activity by NPY. Interestingly, in terms of NPY's coordination of body weigh and bone mass, NPY expression has been identified in both osteoblasts and osteocytes [10], [20]. Moreover, this expression was reduced following exposure of cultured osteoblasts to fluid sheer stress, indicating the potential for a local, load-responsive regulation of NPY. In this manner, increasing load would decrease NPY expression, thereby reducing the tonic repression of osteoblast activity as body weight increased. Thus central efferent signals may provide a systemic regulatory influence, via Y2 receptor action in the arcuate nucleus of the hypothalamus, whilst osteoblastic Y1 receptor action may enable local fine-tuning of the systemic response (Figure 6a).

Taken together, these data highlight the critical role of NPY in the co-ordinated regulation of skeletal tissue. We propose that in situations where hypothalamic NPY expression is decreased, as in short-term overfeeding in which serum leptin levels rise and in turn reduces central NPY signaling [12], [15], bone formation is stimulated as was the case in *NPY ^−/−^* mice. Moreover, when central NPY levels are elevated as in negative energy balance, bone formation is inhibited as was the case in rAAV-NPY+ injected mice. While hypothalamic NPY is ideally placed to modify bone mass to match changes in body weight induced by altered energy homeostasis, NPY produced in bone itself may play an important role in the regulation of bone mass. This later circuit offers also an attractive target to modulate and opens the possibility to increase bone mass without affecting central NPY controlled pathways.

## Materials and Methods

### Generation of NPY ^−/−^ Mice

The generation of the *NPY ^−/−^* mice was described previously [21]. All research and animal care procedures were approved by the Garvan Institute/St Vincent's Hospital Animal Experimentation Ethics Committee and were in agreement with the Australian Code of Practice for the Care and Use of Animals for Scientific Purpose.

### Viral Vector Injection

Elevation of NPY expression was achieved following procedures reported previously [8]. Briefly, 10–12 week-old wild type or *NPY ^−/−^* mice were anesthetized and injected in the PVN with recombinant adeno-associated virus expressing either NPY (rAAV-NPY+) or an empty cassette (rAAV-empty) using a stereotaxic table (David Kopf, California, USA). Brain injection co-ordinates relative to Bregma were posterior, 2.1 mm; lateral, ±0.4 mm; ventral, 5.3 mm corresponding to the PVN [22]. An additional control group was injected with rAAV-NPY+ or rAAV-empty vector in the hippocampus (Co-ordinates relative to Bregma were posterior, 1.7 mm; lateral, ±0.8 mm; ventral, 2.2 mm). One microlitre of either rAAV-NPY+ or rAAV-empty virus (1×10^9^ Pfu/µl) was injected bilaterally over 10 minutes using a 26 gauge guide cannula and a 33 gauge injector (PlasticsOne, Roanoke, USA) connected to a Hamilton Syringe and a syringe infusion pump (World Precision Instruments, Sarasota, Florida). The recombinant AAV expressing NPY was under the control of a neuron specific enolase promoter (rAAV-NPY+), which confined the expression to nerve cells. Mice were housed individually for the ensuing 28 days, with *ad libitum* access to standard chow and water. The efficacy of viral NPY expression was confirmed by the resultant weight gain, a characteristic response to elevated hypothalamic NPY production [8].

### Activity Measurements

Ambulatory activity of individually housed mice was evaluated within the metabolic chambers using an OPTO-M3 sensor system (Columbus Instruments, Columbus, OH, USA), whereby ambulatory counts were a record of consecutive adjacent photo-beam breaks. Cumulative ambulatory counts of X, Y and Z directions were recorded every minute and summed for 1-h intervals.

### Bone Histomorphometry

Mice were injected with the fluorescent compound calcein (Sigma Chemical Company, St Louis, USA) at 20 mg/kg (s.c.) at 10 and 3 days prior to collection. At 14–16 weeks of age, WT and *NPY ^−/−^* mice were sacrificed by cervical dislocation between 10.00–14.00 h for collection of trunk blood in heparinized tubes. Both femora and the lumbar spine were excised and fixed in 4% paraformadehyde for 16 h at 4°C. The right femur was bisected transversely at the midpoint of the long axis and the distal half embedded undecalcified in methacrylate resin (Medim-Medizinische Diagnostik, Giessen, Germany). Similarly, the 4^th^ lumbar vertebrae was defleshed and embedded. Sagittal sections with 5 µm thickness were analysed as previously described [11]. Mineralized bone in the sections was visualized by von Kossa stain. Cancellous bone volume, trabecular thickness and number were calculated. The mineralizing surface (MS, %), mineral apposition rate (MAR, µm/d) and bone formation rate (BFR = MS/BS * MAR, µm2/ µm/d) were calculated as previously described [8], following fluorescence microscopy (Leica, Heerbrugg, Switzerland). Osteoclast surface and osteoclast number were estimated in tartrate-resistant acid phosphatase stained sections where only multinucleated, TRAP-positive cells were included in the analysis. Cortical mineral apposition rate was measured on the anterior periosteal surface in a region extending 1000 µm distal from the mid point of the shaft and in an endosteal region extending 1000 mm proximal from the posterior aspect of the growth plate as previously described [8].

### Bone Densitometry

Whole body bone mineral content (BMC), bone mineral density (BMD), lean mass and fat mass were measured on mice ventral side down with head exclusion and tail inclusion, using a dedicated mouse dual X-ray absorpiometer (DXA) (Lunar Piximus II, GE Medical Systems, Madison WI). Whole femoral BMC and BMD were measured in excised left femora. Femora were scanned with tibiae attached and the knee joint in flexion to ninety degrees to ensure consistent placement and scan of the sagittal profile.

### Micro Computed Tomography

Cancellous bone architecture was evaluated at the distal femoral metaphysis [23], [24]using micro-computed tomography (Micro-CT40, Scanco Medical AG, Basserdorf Switzerland), employing a 12-µm isotropic voxel size. Bone volume fraction (BV/TV, %) was computed without assumptions regarding the underlying bone architecture [25].

### Quantitative Computed Tomography (QCT)

QCT was used to isolate cortical bone for analysis in male mice, using a Stratec XCT Research SA (Stratec Medizintechnik, Pforzheim, Germany). Scans were conducted on excised left femurs as previously described [9] with following settings: voxel size of 70 µm, scan speed of 5 mm/sec and slice width of 0.2 mm. Bones were scanned in 2 consecutive slices, 7 and 7.5 mm from the distal margin of the femur, representing a mid femoral aspect.

### In-Situ Hybridisation of Bone and Hypothalamic Tissue

Antisense and sense riboprobes for mouse NPY were generated from a cDNA region corresponding to bases −72 to +89 of the coding sequence and subcloned into pGEM-T Easy plasmid vector (Promega). Probes were labelled with digoxigenin-UTP by *in vitro* transcription with either SP6 (antisense probe) or T7 (sense probe) RNA polymerase using a DIG RNA labelling kit (Roche) according to the manufacturer's instructions and .purified using Sephadex G25 spin columns and their yield was assessed by dot blot.

*In situ* hybridisation was carried out as previously described with minor modifications [26] on 5 µm sections of decalcified sagittal section of the distal femur. All procedures were carried out at room temperature unless specified otherwise. Sections were dewaxed in xylene, rehydrated through decreasing concentrations of ethanol (100% to 70%), and then fixed in 2% paraformaldehyde in PBS on ice for 10 minutes. After washing in PBS, sections were incubated at 37°C with proteinase K (Roche) at a concentration of 5 µg/mL in 50 mM Tris-HCl (pH 7.5) and 5 mM EDTA for 20 minutes, followed by 0.1 M glycine in PBS for 5 minutes. Sections were acetylated with 1X triethanolamine containing 0.25% (v/v) acetic anhydride for 10 minutes and washed in PBS before hybridisation.

Hybridisation was carried out overnight at 45°C in a humid environment. Sections were incubated with hybridisation solution containing 50% formamide, 5X SSC, 250 µg/ml salmon sperm DNA, 250 µg/ml yeast tRNA, 1x Denhardt's solution, 10% dextran sulphate, 10 mM DTT and 200 ng/mL of digoxigenin-labelled riboprobe. The sections were washed in 2X SSC for 5 minutes followed by 0.2X SSC at 62°C for 30 minutes and 0.1X SSC at 65°C for 30 minutes. They were then incubated with 20 µg/mL RNase A (Roche) in 0.5 M NaCl, 10 mM Tris-HCl, and 1 mM EDTA at 37°C for 15 minutes followed by washes with 2X SSC for 5 minutes, 0.1X SSC at 37°C for 30 minutes. Following the washes, antidigoxigenin Fab antibody fragments conjugated with alkaline phosphatase (Roche) and NBT/BCIP stock solution (Roche) containing 1 mM levamisole were used for colorimetric detection following the manufacturer's instructions.

### Isolation and Differentiation of Bone Marrow Stromal Cells

The isolation and differentiation of plastic adherent bone marrow stromal cells (BMSCs) from 5 to 9 week old male mice was carried out as previously described [10] with minor modifications. Following sacrifice by cervical dislocation, marrow was flushed from femurs and tibias with control media and cells were plated at a density of 1.9×10^6^ cells/cm^2^ in 50 cm^2^ plastic tissue culture plates. Control media consisted of α-minimum essential medium containing 10% fetal bovine serum, 2 mM L-glutamine, 2.2 g/L sodium bicarbonate, 0.017 M HEPES, 100 µg/mL penicillin/streptomycin and 34 mg/L gentamycin. The majority of non-adherent cells were removed by medium changes at 3 and 5 days later and discarded. After 7 days in culture, cells were trypsinised, counted and re-plated at 3×10^4^ cells/cm^2^ in 24-well plates in control media. From this time onwards media was changed 3 times per week.

Differentiation into mineral-producing osteoblasts was achieved by culturing the cells in osteogenic media consisting of control media supplemented with 50 mg/L ascorbic acid and 10 mM β-glycerophosphate. Mineralisation of extracellular matrix was visualised by von Kossa staining with 2% silver nitrate under UV light for 30 minutes. The extent of mineralisation was quantified using the Leica QWin imaging system (Leica Micro-systems, Heerbrugg, Switzerland).

### RNA Extraction and Quantitative Real-Time PCR

Following sacrifice by cervical dislocation, femurs and tibias from 6–10 week old male mice were cleaned and snap frozen in liquid nitrogen. Subsequently femurs and tibias from each leg were homogenised separately in TRIzol® reagent using a polytron. RNA extractions were carried out using TRIzol® reagent according to the manufacturer's instructions. RNA samples were checked for quality and quantified using the Agilent 2100 Bioanalyser (Agilent Technologies) according to the manufacturer's instructions. One microgram of total RNA was taken for cDNA synthesis with oligo(dT)_20_ and random hexamers using the SuperScript III First-Strand Synthesis System for reverse transcription-PCR (Invitrogen). Quantitative real-time PCR was then carried out using the TaqMan Universal PCR master mix (Applied Biosystems), ABI Prism 7900 HT Sequence Detection System and Software and inventoried kits containing primers and probes from Applied Biosystems. To control for variability in amplification due to differences in starting mRNA concentrations, GAPDH was used as an internal standard. The relative expression of target mRNA was computed from the target Ct values and the GAPDH Ct value using the standard curve method (Sequence Detection Systems Chemistry Guide, Applied Biosystems).

### Statistical Analyses

Dual genotype comparisons were assessed using two-tailed students T-test. Multiple genotype comparisons were assessed by factorial ANOVA followed by Fisher's or Contrasts post-hoc tests, using StatView version 4.5 or Super-ANOVA (Abacus Concepts Inc, CA, USA). For all statistical analyses, *P*<0.05 was accepted as being statistically significant.

## References

[1] Moore RE, Smith CK, Bailey CS, Voelkel EF, Tashjian AH (1993). Characterization of beta-adrenergic receptors on rat and human osteoblast-like cells and demonstration that beta-receptor agonists can stimulate bone resorption in organ culture.. Bone Miner.

[2] Kellenberger S, Muller K, Richener H, Bilbe G (1998). Formoterol and isoproterenol induce c-fos gene expression in osteoblast-like cells by activating beta2-adrenergic receptors.. Bone.

[3] Takeda S, Elefteriou F, Levasseur R, Liu X, Zhao L (2002). Leptin regulates bone formation via the sympathetic nervous system.. Cell.

[4] Stanley BG, Kyrkouli SE, Lampert S, Leibowitz SF (1986). Neuropeptide Y chronically injected into the hypothalamus: a powerful neurochemical inducer of hyperphagia and obesity.. Peptides.

[5] Erickson JC, Hollopeter G, Palmiter RD (1996). Attenuation of the obesity syndrome of ob/ob mice by the loss of neuropeptide Y.. Science.

[6] Baldock PA, Sainsbury A, Couzens M, Enriquez RF, Thomas GP (2002). Hypothalamic Y2 receptors regulate bone formation.. J Clin Invest.

[7] Blomqvist AG, Herzog H (1997). Y-receptor subtypes–how many more?. Trends Neurosci.

[8] Baldock PA, Sainsbury A, Allison S, Lin EJ, Couzens M (2005). Hypothalamic control of bone formation: distinct actions of leptin and y2 receptor pathways.. J Bone Miner Res.

[9] Baldock PA, Allison S, McDonald MM, Sainsbury A, Enriquez RF (2006). Hypothalamic regulation of cortical bone mass: opposing activity of Y2 receptor and leptin pathways.. J Bone Miner Res.

[10] Baldock PA, Allison SJ, Lundberg P, Lee NJ, Slack K (2007). Novel role of Y1 receptors in the coordinated regulation of bone and energy homeostasis.. J Biol Chem.

[11] Allison SJ, Baldock P, Sainsbury A, Enriquez R, Lee NJ (2006). Conditional deletion of hypothalamic Y2 receptors reverts gonadectomy-induced bone loss in adult mice.. J Biol Chem.

[12] Ducy P, Amling M, Takeda S, Priemel M, Schilling AF (2000). Leptin inhibits bone formation through a hypothalamic relay: a central control of bone mass.. Cell.

[13] Elefteriou F, Takeda S, Liu X, Armstrong D, Karsenty G (2003). Monosodium glutamate-sensitive hypothalamic neurons contribute to the control of bone mass.. Endocrinology.

[14] Reid IR (2008). Relationships between fat and bone.. Osteoporos Int.

[15] Sipols AJ, Baskin DG, Schwartz MW (1995). Effect of intracerebroventricular insulin infusion on diabetic hyperphagia and hypothalamic neuropeptide gene expression.. Diabetes.

[16] Heilig M, Vecsei L, Widerlov E (1989). Opposite effects of centrally administered neuropeptide Y (NPY) on locomotor activity of spontaneously hypertensive (SH) and normal rats.. Acta Physiol Scand.

[17] Sample SJ, Behan M, Smith L, Oldenhoff WE, Markel MD (2008). Functional adaptation to loading of a single bone is neuronally regulated and involves multiple bones.. J Bone Miner Res.

[18] Hamrick MW, Ding KH, Ponnala S, Ferrari SL, Isales CM (2008). Caloric restriction decreases cortical bone mass but spares trabecular bone in the mouse skeleton: implications for the regulation of bone mass by body weight.. J Bone Miner Res.

[19] Reid IR, Cornish J, Baldock PA (2006). Nutrition-related peptides and bone homeostasis.. J Bone Miner Res.

[20] Igwe JC, Jiang X, Paic F, Ma L, Adams DJ (2009). Neuropeptide Y is expressed by osteocytes and can inhibit osteoblastic activity.. J Cell Biochem.

[21] Karl T, Duffy L, Herzog H (2008). Behavioural profile of a new mouse model for NPY deficiency.. Eur J Neurosci.

[22] Franklin KBJ, Paxinos G (1997). The Mouse Brain in Stereotaxic Coordinates..

[23] Bouxsein ML, Pierroz DD, Glatt V, Goddard DS, Cavat F (2005). beta-Arrestin2 regulates the differential response of cortical and trabecular bone to intermittent PTH in female mice.. J Bone Miner Res.

[24] Ferrari SL, Pierroz DD, Glatt V, Goddard DS, Bianchi EN (2005). Bone response to intermittent parathyroid hormone is altered in mice null for {beta}-Arrestin2.. Endocrinology.

[25] Hildebrand T, Laib A, Muller R, Dequeker J, Ruegsegger P (1999). Direct three-dimensional morphometric analysis of human cancellous bone: microstructural data from spine, femur, iliac crest, and calcaneus.. J Bone Miner Res.

[26] Parker RM, Herzog H (1999). Regional distribution of Y-receptor subtype mRNAs in rat brain.. Eur J Neurosci.

